# Clinical utility and applicability of circulating tumor DNA testing in esophageal cancer: a systematic review and meta-analysis

**DOI:** 10.1093/dote/doab046

**Published:** 2021-07-21

**Authors:** Swathikan Chidambaram, Sheraz R Markar

**Affiliations:** Department of Surgery and Cancer, Imperial College London, London, UK; Department of Surgery and Cancer, Imperial College London, London, UK; Department of Molecular Medicine and Surgery, Karolinska Institutet, Stockholm, Sweden

**Keywords:** esophageal adenocarcinoma, esophageal cancer, esophageal squamous cell cancer

## Abstract

Esophageal cancer is an aggressive malignancy with a relatively poor prognosis even after multimodality therapy. Currently, patients undergo a series of investigations that can be invasive and costly or pose secondary risks to their health. In other malignancies, liquid biopsies of circulating tumor DNA (ctDNA) are used in clinical practice for diagnostic and surveillance purposes. This systematic review summarizes the latest evidence for the clinical applicability of ctDNA technology in esophageal cancer. A systematic review of the literature was performed using MEDLINE, EMBASE, the Cochrane Review and Scopus databases. Articles were evaluated for the use of ctDNA for diagnosis and monitoring of patients with esophageal cancer. Quality assessment of studies was performed using the QUADAS-2 tool. A meta-analysis was performed to assess the diagnostic accuracy of sequencing methodologies. We included 15 studies that described the use of ctDNA technology in the qualitative synthesis and eight studies involving 414 patients in the quantitative analysis. Of these, four studies assessed its utility in cancer diagnosis, while four studies evaluated its use for prognosis and monitoring. The pooled sensitivity and specificity for diagnostic studies were 71.0% (55.7–82.6%) and 98.6% (33.9–99.9%), while the pooled sensitivity and specificity for surveillance purposes were 48.9% (29.4–68.8%) and 95.5% (90.6–97.9%). ctDNA technology is an acceptable method for diagnosis and monitoring with a moderate sensitivity and high specificity that is enhanced in combination with current imaging methods. Further work should demonstrate the practical integration of ctDNA in the diagnostic and surveillance clinical pathway.

## INTRODUCTION

Esophageal cancer is an aggressive cancer with a mean estimated five-year survival rate of just 35–45% even after treatment with curative intent.^1^^,^^2^ As early stage cancer is asymptomatic, most patients present late with advanced disease, and within this cohort, the reported survival rate drops further to approximately 5–10%.^3^ Aggressive tumor biology associated with a high prevalence of disease recurrence often further lower the prognosis.^4,5^ Currently, patients undergo a series of diagnostic and staging investigations, including computed tomography scans (CT scan), positron emission tomography (PET) scans, endoscopic ultrasound (EUS) and endobronchial ultrasound (EBUS).^6^ While these investigations are highly accurate in overt disease, they have limited value in detecting early disease or are too invasive to be used at an early stage of the diagnostic pathway.^7^^,^^8^ Unlike colorectal, hepatocellular or pancreatic cancers, there is no reliable biomarker that can be tested and tracked noninvasively for diagnostic or surveillance purposes in esophageal cancer.^9^^,10^ Consequently, patients typically undergo a series of the aforementioned investigations for diagnosis, detection of recurrence and response to treatment, which may lead to additional unnecessary morbidity.^11^ Recently, there has been more work focusing on the use of circulating tumor DNA (ctDNA) in both the diagnostic and surveillance setting.

Cell-free DNA (cfDNA) refers to any circulating DNA present in the bloodstream and can be derived from both cancerous and normal cells.^12^ ctDNA refers to shorter sequences of fragmented DNA derived specifically from cancer cells and present freely in the bloodstream.^13^^,^^14^ As cancers grow, they release ctDNA that can be detected in peripheral vasculature. ctDNA forms about <0.01% to 10% of cfDNA, and the exact proportion varies with time depending on the primary tumor, tumor grade and vascularity, physiological clearance (liver or kidney disease), rate of release, cell status, time of blood draw and ongoing therapies. Nevertheless, advances in genome sequencing, including targeted methods such as next-generation sequencing (NGS) and deep sequencing, have made analysis of ctDNA more feasible. This has led to its use in the diagnosis and monitoring of malignancies, including bladder, breast, colorectal, esophageal, gastric, lung and pancreatic cancers.^15^ Its utility has been best validated for non–small cell lung cancer and breast cancer, including its use in diagnosis, detection of recurrence and treatment response in clinical trials such as the AURA3 trial, the ASSESS trial and the INSPIRE study.^16–18^ At the time of writing, there is still no up-to-date work on the evidence for the use of ctDNA specifically in esophageal cancer.^19^ This systematic review and meta-analysis aims to summarize the latest evidence for the clinical applicability of ctDNA technology in esophageal cancer and address its current challenges.

## METHODS

Literature search methods, inclusion and exclusion criteria, outcome measures and statistical analysis were defined according to the Preferred Reporting Items for Systematic Reviews and Meta-Analyses (PRISMA).^20^ Patients were not involved in the conception, design, analysis, drafting, interpretation or revision of this research. Hence, ethical approval was not required and thus not sought for this study.

### Literature search

The following databases were searched: MEDLINE (1946 until the first week of February 2021) via OvidSP; MEDLINE in-process and other nonindexed citations (latest issue) via OvidSP; Ovid EMBASE (1974 to latest issue); and Scopus (1996 till present). The last search was performed on February 2021. Search terms used several strings that were linked by standard modifiers in the following order: ‘ctDNA’, ‘circulating tumor DNA’ OR ‘liquid biopsy’ as well as ‘esophageal cancer’, ‘esophageal squamous cell cancer’, ‘esophageal adenocarcinoma’, ‘ESCC’, ‘EAC’ or ‘esophageal malignancy’. Additionally, the references of included articles were hand-searched to identify any additional studies.

### Selection and quality assessment of studies

Studies were screened for inclusion by both authors (SC and SRM). Studies were included if they had investigated the use of ctDNA as a method for evaluating both esophageal squamous cell cancer and adenocarcinoma. Studies with diagnostic, prognostic and monitoring intents were included. Studies were excluded if they did not evaluate sequencing technologies involving ctDNA; did not report outcomes of DNA sequencing method; did not involve samples obtained from patients; had incomplete data on outcome measures; were not in the English language or had incompatible designs including conference abstracts, letters, comments and reviews. Studies were assessed for robustness of methodology using the quality assessment tool for diagnostic accuracy studies 2 (QUADAS-2). The QUADAS-2 comprises four domains covering patient selection, index test, reference standard and flow of patients through the study and timing of the index test(s) and reference standard. Each domain is evaluated in terms of the risk of bias, and the first three are also assessed for any concerns regarding applicability. In doing so, it highlights aspects of the study design that may be exposed to bias.

### Statistical analysis

All statistical analyses were performed using STATA/SE, version 16.0 (StataCorp LLC, College Station, TX). The overall pooled estimate of sensitivity and specificity with their corresponding 95% confidence interval (95% CI) was calculated using the random-effects model by the command in STATA/SE. Sensitivity was defined as the proportion of patients with esophageal cancer that were correctly confirmed by detectable ctDNA, while specificity was defined as correctly identifying patients without the disease. Forest plots were used to visualize the variation of the diagnostic parameters effect size estimates with 95% CI and weights from the included studies.

## RESULTS

### Study selection

**Fig. 1:**
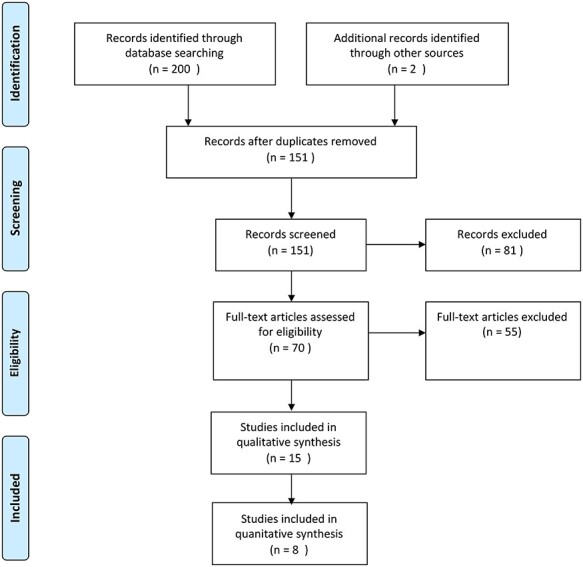
PRISMA flow diagram for study selection.

The database search yielded a total of 200 studies. Of these, 49 duplicates were removed. Titles and abstracts of the remaining 151 studies were assessed for eligibility, and 81 studies were removed. A further 55 studies were excluded after full-text review due to incompatible outcome measures; study design or small sample sizes of less than 20 specimens (Fig. 1).^21^ Fifteen studies had described the use of ctDNA technology in the diagnosis and monitoring of esophageal cancer (Table 1).^22–33^

**Table 1.** The use of ctDNA for diagnosis and monitoring

Study	Sequencing method	Purpose	No. of patients	No. of samples	Sensitivity	Specificity
Bettegowda *et al.*	Agilent SureSelect (targeted, 100 genes)	Diagnostic	21	–	–	–
Davidson *et al.*	Illumina HiSeq2500 (targeted)	Diagnostic	24	24	–	–
Jia *et al*.	Illumina HiSeq3000 Sequencing System (targeted, 180 gene panel)	Diagnostic	25	69	71.4	50
Schrock *et al*.	Illumina HiSeq2500 or 4000 (targeted, 62 genes(	Diagnostic	56	–	–	–
Ueda *et al.*	HiSeq2000 (targeted, 53 genes)	Diagnostic	13	64	78.9	100
Maron *et al*.	Guardant360 test (targeted)	Diagnostic	1630	2140	–	–
Riviere *et al.*	Next-generation sequencing (targeted, 68 genes)	Diagnostic	8	–	–	–
Komatsu *et al.*	RT-PCR	Diagnostic	103	–	69.8	80.0
Andolfo *et al.*	ABI PRISM 7900HT Sequence Detection (RT-PCR detection of erbB2 and B-actin genes)	Surveillance	41	–	80	95
Boniface *et al*.	Dual-Index Degenerate Adaptor-Sequencing (targeted)	Surveillance	3	–	–	–
Ko *et al.*	Qubit dsDNA HS Assay Kit	Surveillance	60	143	45.5	89.5
Ococks *et al.*	NextSeq 550 (targeted, 77 genes)	Surveillance	97	245	35	97
Openshaw *et al*.	Next-generation sequencing (targeted, 4 genes)	Surveillance	35	116	85.7	100
Azad *et al.*	CAPP-seq (Deep sequencing, 607 genes)	Diagnostic and Surveillance	40	218	71.4 (100% if combined with PET-CT)	100 (100% if combined with PET-CT)
Luo *et al.*	Illumina TruSight Cancer sequencing (targeted,	Diagnostic and Surveillance	11	55	–	–

### Quality appraisal

Assessment of studies using the QUADAS-2 tool showed that studies were of a high quality (Table 2). The risk of bias and concerns on their applicability was low across most domains. Some risk of bias was present due to the heterogeneity of the patients included, and analysis of diagnostic accuracy was not available for some seven studies that were not included in the meta-analysis.

**Table 2.** QUADAS assessment of included studies

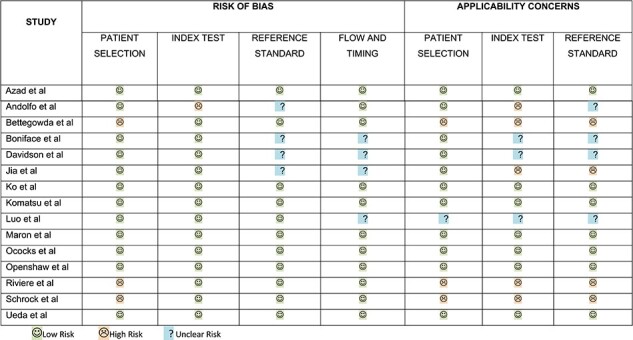

### ctDNA as a diagnostic and surveillance tool: qualitative analysis

Of the 15 studies included, eight studies focused on the diagnostic potential of ctDNA; five studies on its use as a surveillance tool and two studies on both intents. Majority of the studies used targeted sequencing methodologies. For example, Ueda *et al*. used next-generation sequencing to amplify the DNA of 53 genes and detected 55 somatic mutations of TP53, FAT3, MLL3 and AJUBA.^34^ Similarly, Luo *et al*. used targeted and whole exome sequencing to detect somatic mutations in 55 samples from 11 ESCC patients.^32^ Two studies had shown the potential of ctDNA as a risk stratification tool for disease severity and recurrence. Jia *et al*. used it as a risk stratification tool by showing that patients with metastasis to lymph nodes (LN) had a higher number of mutations than patients without LN metastasis.^31^ In their longitudinal studies, both Openshaw *et al*. and Ococks *et al*. serially sampled peripheral blood to show ctDNA can be predictive of relapses following surgery.

Three studies had included other types of cancers, of which esophageal cancer comprised a small minority. For example, Bettegowda *et al*. used PCR to amplify and detect ctDNA in more than 75% of late-stage cancers, including gastroesophageal cancer.^28^ Between all cancers, Schrock *et al*. reported a 63% match between ctDNA and tissue samples, including alterations in TP53 (72%), KRAS (35%), PIK3CA (14%), BRAF (8%) and EGFR (7%).^24^ Riviere *et al*. reported alterations in the same genes in their analysis.^33^ In terms of monitoring, Luo *et al*. compared the plasma of patients before and after surgery and report that the allele frequency of mutated ctDNA was much lower postsurgery, suggesting that surgery reduced the tumor burden.^32^ In another study of 40 patients by Andolfo *et al*., patients with esophageal cancer had a higher number of copy number variations of the erbB2 gene that was associated with a worse prognosis.^29^ Ueda *et al*. identified somatic mutations from both primary and recurrent tumours.^34^ Specifically, they detected an increased allele frequency in ctDNA six months earlier than tumor recurrences were detected on imaging, a finding reflected by studies involving other cancer types.^35^ Davidson *et al*. showed that factors such as the presence of liver metastases were associated with a high ctDNA fraction, which subsequently correlated with a poorer survival outcomes.

### ctDNA as a diagnostic and monitoring tool: quantitative analysis

**Fig. 2:**
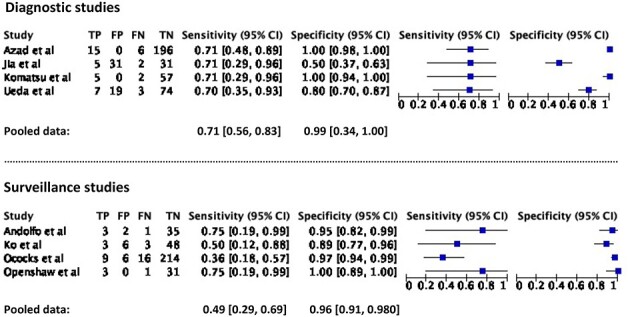
Pooled sensitivity and specificity for diagnostic and prognostic studies.

**Fig. 3:**
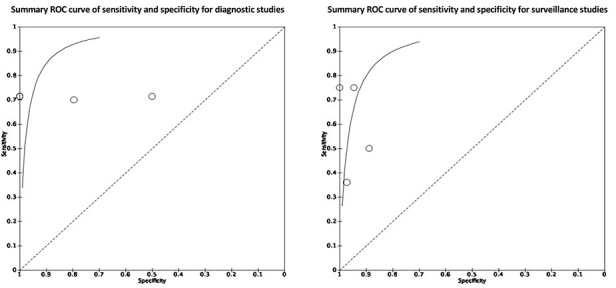
Summary ROC curves for diagnostic and prognostic studies.

Eight studies involving four hundred and fourteen patients provided sufficient data of true-positive, true-negative, false-positive and false-negative rates for the calculation of sensitivity and specificity.^25–27^^,^^29^^,^^31^^,^^34^^,^  ^36^^,^^37^ Of these, four studies assessed its utility in cancer diagnosis, while four studies evaluated its use for prognosis and monitoring (Table 1). The pooled sensitivity and specificity for diagnostic studies were 71.0% (55.7–82.6%) and 98.6% (33.9–99.9%), while the pooled sensitivity and specificity for monitoring purpose studies were 48.9% (29.4–68.8%) and 95.5% (90.6–97.9%), as visualized on the forest plots and summary ROC curves (Figs 2 and 3).

## DISCUSSION

This systematic review and meta-analysis is the first to provide updated evidence on the accuracy, scalability and applicability of ctDNA testing for diagnosis and surveillance of esophageal cancer. It also addresses the downsides of the methodology and draws lessons from its use in breast and lung cancers that can be extended to overcome these challenges. Our review of 15 articles demonstrates that ctDNA testing can be used to detect patients with cancer; differentiate between different stages and grades of esophageal cancers; characterize the molecular heterogeneity present within and between patients; assess treatment response and monitor tumor progression. Pooled statistical analysis of 414 patients highlights that ctDNA is a robust, reliable and feasible method with high specificity and moderate sensitivity for both diagnosis and surveillance purposes.

The use of ctDNA has been more extensively explored in lung cancer and breast cancer as they have well-established genetic mutations that drive the carcinogenesis pathways. Unlike CT abdomen or EUS, ctDNA does not provide anatomical confirmation but our study shows its potential as a screening or confirmatory adjunct. More importantly, the uniqueness of ctDNA as a diagnostic modality stems from its ability to characterize cancers at a molecular level in a noninvasive manner. A major aspect of tumor biology is that cancers evolve depending on their tumor microenvironment (TME) by acquiring genetic and epigenetic alterations in response to the TME and the associated stressors. This generates different clones of cancer cells within the same patient and is termed tumor heterogeneity, indicating that no two tumor cells are the same at the molecular level. Heterogeneity is a reason why the response to therapies differs between patients.^38^ This will be especially critical in stage III and IV cancers where a greater diversity of mutations is often seen.^39^ Several of the included studies identified different alterations of ctDNA and thus detected tumor heterogeneity, differentiated between different disease severity and were able to assess changes in heterogeneity after patients had undergone treatments.^26^^,^^31^^,^^40^^,^^41^ A liquid biopsy of ctDNA can capture a snapshot of heterogeneity and thus aid in delivering treatments tailored to specific mutations, especially with the advent of molecular therapies for esophageal cancer.^42^

From a monitoring perspective, an effective testing modality should be able to detect tumor response to treatment.

Ueda *et al*. reported an increased allele frequency in ctDNA 6 months earlier than tumor recurrences were detected on imaging, a finding reflected by studies involving other cancer types.^34^^,^^35^ Clinically, 6 months is a crucial window during which surgical and nonsurgical interventions can be used to gain control over tumor recurrence. Furthermore, the allele frequency of specific mutations such as TP53 had a stronger association with tumor burden than conventional biomarkers of ESCC. Besides surgery, ctDNA was also shown to be less in patients who have undergone radiotherapy, which correlated with a poorer progression free survival, overall survival and formation of distant metastases. A larger proportion of patients with tumor progression also had new mutations in their plasma after chemoradiotherapy than patients without progression.^27^ In a more recent larger study of 97 patients treated with neoadjuvant chemotherapy and surgery, the authors analyzed using a pan-cancer ctDNA panel comprising of 77 genes from 245 samples and showed that ctDNA can be reliably used to detect residual disease postintervention, recurrence and stratify patients according to their survival.^25^ Notably, their analysis also picked up clonal hematopoiesis with indeterminate potential (CHIP), which further adds to the validity of the analytical method.

### Challenges for clinical applications of ctDNA testing

The practical aspects of ctDNA analysis raised several challenges to its scalability.^43^ cfDNA has a short half-life of approximately 16 minutes and thus needs prompt stabilization in either EDTA or cell-stabilizing tubes.^44^ If EDTA tubes are used, the sample must be processed within 6 hours to avoid lysis of white cells, which can further dilute the ctDNA fraction. Thus, there is a time-sensitive element to how the sample is collected and handled, similar to cerebrospinal fluid analysis for xanthochromia. As mentioned, there are several sequencing methods available to amplify the small fraction of ctDNA, and the assessment of ctDNA technology is indirectly an assessment of which sequencing platform is used. Depending on the method used, the end-result may vary and will affect its interpretation. Preanalytical considerations include tumor-level factors (tumor type, metastatic sites, stage of disease, tumor heterogeneity, clonal versus subclonal variants); patient factors (age, gender and comorbidities such as liver or kidney disease) and technical factors (time of draw, type of sample drawn). Thus, the sequencing technology and its pre-analytical aspects need to be standardized across testing centers to ensure their accuracy, similar to how the NEQAS ensures the validity of routine laboratory tests.^45–48^ This will enable the majority of this process to be automated and thus be used on a large scale.

The 5-year survival rate for esophageal cancer can be as low as 5%, and this is partly due to the insidious progression of the cancer.^3^ Accurate diagnosis of esophageal cancer is vital to provide patients with optimal and effective treatment options, and appropriate prognostication. Current diagnostic strategies include conventional CT imaging and invasive methods such as endoscopy, endoscopic ultrasound, endobronchial ultrasound and CT-guided biopsies. A main advantage of ctDNA testing is that it is a noninvasive method for diagnosing esophageal cancer. Solid tissue biopsies are tested for mutations of HER2, PDL1, microsatellite instability (MSI) genes and mismatch repair genes such as MLH1, MSH2, MSH6 and PMS2. Hence, although these investigations can be laborious for the patient, this pathway ensures a high detection rate among symptomatic patients up to 79% to 100%.^49^ For ctDNA to be widely adopted, it has to match an equivalent detection rate.

Based on previous work, the usefulness of ctDNA for clinical purposes is highest for lung cancer.^50^ That lies in the strong research groundwork that had identified specific mutations driving the cancers and subsets of patients with variations of these mutations. This gave diagnostic testing a specific range of definitive targets to analyze and made it more focused. Furthermore, treatments for lung cancer are aimed at specific targets and thus have become personalized at a molecular level. For example, ctDNA analysis can be focused toward picking up the incidence of not only the epidermal growth factor receptor (EGFR) gene mutations but also specific variations of it such as the pT790M mutation. This allows for patients to be treated with tyrosine kinase inhibitors such as Osimertinib, leading to a personalized therapeutic approach.^51^ In esophageal cancer, the commonest mutations such as p53 and KRAS are also the drivers of tumorigenesis in other cancers, including gastrointestinal malignancies.^52^^,^^53^ Furthermore, many of the described mutations that characterize esophageal cancer, such as TP53 and CDKN2A, also occur in precursor lesions such as Barrett’s esophagus and high-grade dysplasia, lowering the specificity of ctDNA testing.^54^ As a result, it is more difficult to deliver tailor made molecular therapies for these patients. Thus, further work is needed in identifying molecular markers that are very prevalent and specific for esophageal cancer in order to make ctDNA measurement clinically useful.

A major challenge to the clinical utility of ctDNA is the costs associated with the necessary infrastructure. As with any developing technology, the initial upfront cost of setting up the equipment and workforce is high. For example, in one study, Abbosh *et al*. calculated that a personalized assay would cost approximatly $1750 even if it were to target a small range of single nucleotide variants.^55^ In another study, Vessies *et al*. compared six different testing platforms for detecting colorectal cancer ctDNA and estimated a cost range of €39–821. Testing platforms such as BEAMing, which are more sensitive and specific than other methods, cost upwards of €486 per sample.^56^ In contrast, studies of ctDNA testing are assessed to be more cost-effective than a solid biopsy. For example, while a ctDNA targeting specific mutations costs £170, the equivalent solid CT-guided biopsy would cost in excess of £1000 even if the costs associated with potential complications are not included. Hence, the costs of ctDNA testing vary with the cancer and the DNA defects associated with it. While studies have reported varying costs per sample, there is a paucity of formal economic health analyses comparing the costs of ctDNA with current alternatives for the commonest malignancies, including breast, lung and colorectal cancer. Currently, there is no study that has evaluated the cost-effectiveness of using ctDNA as a diagnostic or prognostic instrument in esophageal cancer. This is especially pertinent to the scalability of ctDNA given that esophageal cancer treatment is an expensive process for the patient and has been estimated to be more costly than other cancers irrespective of the stage.^57^^,^^58^

**Table:** MOOSE checklist for meta-analyses of observational studies

Item no	Recommendation	Reported on page no
Reporting of background should include		
1	Problem definition	3
2	Hypothesis statement	–
3	Description of study outcome(s)	4
4	Type of exposure or intervention used	4–5
5	Type of study designs used	4–5
6	Study population	5
Reporting of search strategy should include		
7	Qualifications of searchers (e.g., librarians and investigators)	Title page
8	Search strategy, including time period included in the synthesis and key words	3–4 (Section 2.1)
9	Effort to include all available studies, including contact with authors	3–4 (Section 2.1)
10	Databases and registries searched	3–4 (Section 2.1)
11	Search software used, name and version, including special features used (e.g., explosion)	3–4 (Section 2.1)
12	Use of hand searching (e.g., reference lists of obtained articles)	3–4 (Section 2.1)
13	List of citations located and those excluded, including justification	8, Table 2, Fig. 1
14	Method of addressing articles published in languages other than English	3–4 (Section 2.1)
15	Method of handling abstracts and unpublished studies	3–4 (Section 2.1)
16	Description of any contact with authors	–
Reporting of methods should include		
17	Description of relevance or appropriateness of studies assembled for assessing the hypothesis to be tested	3–4
18	Rationale for the selection and coding of data (e.g., sound clinical principles or convenience)	3–4
19	Documentation of how data were classified and coded (e.g., multiple raters, blinding and interrater reliability)	–
20	Assessment of confounding (e.g., comparability of cases and controls in studies where appropriate)	–
21	Assessment of study quality, including blinding of quality assessors, stratification or regression on possible predictors of study results	3–4 Table 2
22	Assessment of heterogeneity	4 Table 2
23	Description of statistical methods (e.g., complete description of fixed or random-effects models, justification of whether the chosen models account for predictors of study results, dose–response models, or cumulative meta-analysis) in sufficient detail to be replicated	4
24	Provision of appropriate tables and graphics	Tables 1–2, Figs 1–3
Reporting of results should include		
25	Graphic summarizing individual study estimates and overall estimate	Figs 2–3
26	Table giving descriptive information for each study included	Table 1
27	Results of sensitivity testing (e.g., subgroup analysis)	Fig. 3 Table 1
28	Indication of statistical uncertainty of findings	5
Reporting of discussion should include		
29	Quantitative assessment of bias (e.g., publication bias)	5–6
30	Justification for exclusion (e.g., exclusion of non-English language citations)	5–6
31	Assessment of quality of included studies	5–6 (QUADAS assessment)
Reporting of conclusions should include		
32	Consideration of alternative explanations for observed results	5–6
33	Generalization of the conclusions (i.e., appropriate for the data presented and within the domain of the literature review)	5–6
34	Guidelines for future research	5–6
35	Disclosure of funding source	–

*From*: Stroup *et al*.^15^ for the Meta-analysis of Observational Studies in Epidemiology (MOOSE) Group. Meta-analysis of observational studies in epidemiology. A proposal for reporting. *JAMA*. 2000;283:2008–2012. doi: 10.1001/jama.283.15.2008.

Our study has several strengths, including its novelty in being the first to quantitatively assess the accuracy of liquid biopsies in esophageal cancer. By stratifying studies based on their intents, we have shown that ctDNA is a robust technology for diagnosis and surveillance. Previous studies have treated ‘liquid biopsy’ as an umbrella term and included the use of cfDNA. Compared to ctDNA, which is solely derived from tumor cells, cfDNA is derived from normal and tumor cells. By considering only ctDNA, our work has assessed the utility of a more specific marker than cfDNA. While our work is comprehensive, this inherently presents several limitations, largely the heterogeneity between the different included studies due to differences in the type, grade and stages of esophageal cancer as well as the ctDNA platforms used. We have estimated the variance in effect size by using a random-effects model to account for the heterogeneity. Furthermore, the meta-analysis consists of small studies, often with little longitudinal follow-up. Our analysis does not stratify the results based on histology, and given that adenocarcinomas and squamous cell carcinomas are two distinct pathologies that behave differently, our study does not delineate this variation of ctDNA testing. Majority of the studies included patients at different times of the patient pathway such as pretreatment, after chemo- or chemoradiation therapy (and the timing after chemo-/chemoradiation) and after esophagectomy, which is an additional confounding factor. This is key given that studies were retrospective and hence predominantly included tumor-informed cases, where samples were obtained from patients with known cancer. Results of further work would be more applicable if tumor-uninformed cases were included, and cancer was confirmed after ctDNA analysis was performed. Taken together, future work should prospectively investigate whether ctDNA is a valid and reliable method for diagnosis and surveillance in tumor-uninformed samples stratified based on their histology over a longer follow-up period. Lastly, while we have evaluated the diagnostic accuracy of the studies, we have not carried out a formal economic analysis to assess its cost-effectiveness. Ultimately, this would prove that ctDNA has a long-term gain in cost–benefit and seal its incorporation into clinical practice.

## CONCLUSION

There is an abundance of work dedicated to the utility of ctDNA in other malignancies, especially breast and lung cancers. Currently, there is a paucity of large-scale studies evaluating its usefulness in esophageal cancer. Prospective studies involving small sample sizes have been used to confirm the use of ctDNA for both diagnosis and monitoring. However, further progression of its clinical applications depends on making this both a cost-effective and scalable option, which depends on ensuring that its accuracy and reliability matches or supersedes current options. This relies on determining the best and most feasible methodology for performing ctDNA analysis and ensuring that this can be standardized across centers. Hence, further work should be aimed in these areas. In an era where oncological treatment is becoming more personalized, the incorporation of ctDNA has great potential if the lessons from management of other cancers were to be extended to esophageal cancer.
